# Properdin deficiency associated with systemic meningococcal disease due to a novel p.Cys337Arg pathogenic variant

**DOI:** 10.1016/j.gendis.2023.101134

**Published:** 2023-10-04

**Authors:** Laura González-Sánchez, Ana Mei Agudo, Anne Van Den Rym, María Isabel Begiristain, Alazne Saizar, Rebeca Pérez de Diego, Pilar Nozal, Alberto López-Lera, Margarita López-Trascasa, Fernando Corvillo

**Affiliations:** a Complement Research Group, Hospital La Paz Institute for Health Research (IdiPAZ), La Paz University Hospital, Madrid 28046, Spain; b Center for Biomedical Network Research on Rare Diseases, Madrid, Spain; c Laboratory of Immunogenetics of Human Diseases, IdiPAZ Institute for Health Research, La Paz University Hospital, Madrid 28046, Spain; d Ormaiztegui Clinic, Guipúzcoa 20216, Spain; e Immunology Unit, La Paz University Hospital, Madrid 28046, Spain; f Departamento de Medicina, Universidad Autónoma de Madrid, Madrid 28029, Spain

Properdin (FP) is a soluble glycoprotein with a key role in the regulation of the alternative pathway (AP) of the complement system.^1^ Neutrophils are the main source of FP, although monocytes, bone marrow progenitors, and T cells also synthesize this complement regulator. FP is codified by the gene *CFP*, located at Xp11.23–p11.3, and formed by 10 exons, of which exons 2 to 10 are translated conforming six thrombospondin type I repeats (TSR1-6).^1^ FP deficiency (MIM # 312060) is a rare X-linked disorder that contributes to increased susceptibility to infectious diseases, mainly caused by rare strains of *Neisseria meningitidis* such as serogroups W-135 and Y. Since its initial description in 1982,^2^ more than 100 cases have been reported worldwide demonstrating susceptibility to meningococcal infections and sepsis.^1^ FP deficiency can be divided into three subtypes: total deficiency (type I), partial deficiency (type II), and deficiency due to a dysfunctional protein (type III). In the present study, we investigated the members of a non-consanguineous Spanish family with a novel *CFP* mutation causing type I FP deficiency.

The index case was a male who suffered meningitis at the age of 15 years (III.1; Fig. 1A). He presented fever (>38 °C), frontal headache and vomiting with no reported cervical stiffness in the first hours. The next day he was admitted to the hospital due to bradypsychia, skin pallor, petechiae on the hands and feet and nuchal rigidity Laboratory investigations revealed leukocytosis (25.06 × 10^3^/μL) and increased levels of procalcitonin (3.91 ng/mL) and C-reactive protein (318 mg/L). Coagulation studies showed an increase in prothrombin time (24 s), fibrinogen (741 mg/dL), and D-dimer (1520 ng/mL). Suspecting meningitis of probable bacterial origin, blood culture and lumbar puncture were requested confirming systemic meningococcal disease caused by *Neisseria meningitidis* serogroup Y. The patient was immediately treated with vancomycin and ceftriaxone with a good clinical response.

**Figure 1:**
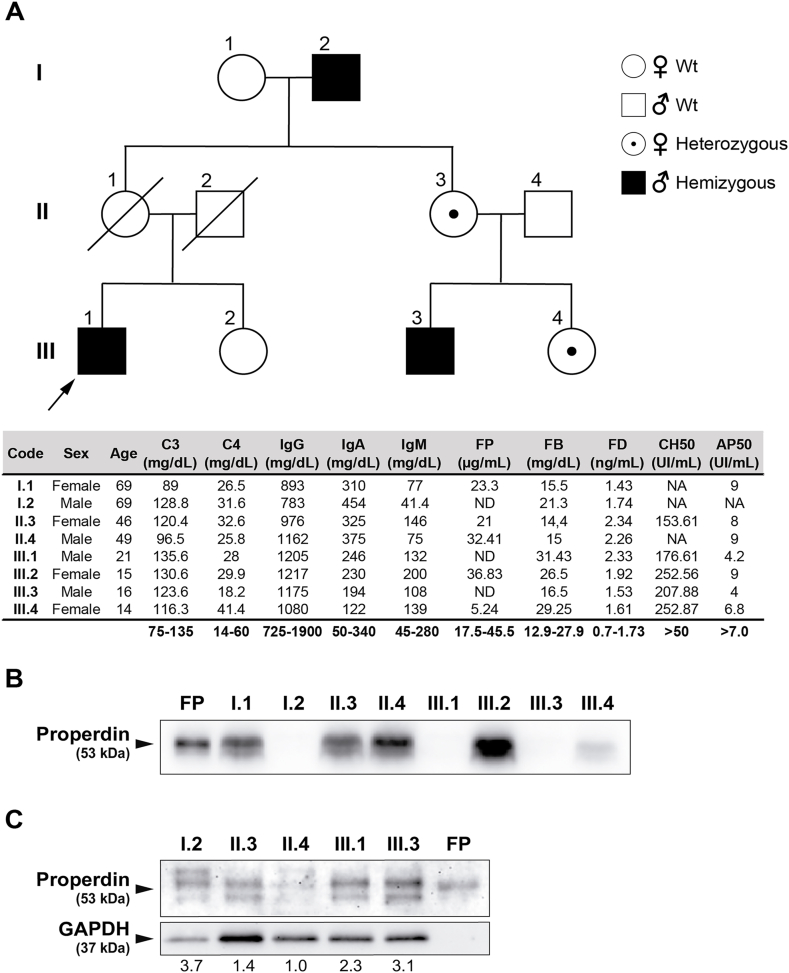
Familial pedigree and Western blotting analysis of plasmatic and intracellular FP. **(A)** The family pedigree with the C337R mutation in the CFP gene. The index patient is marked by an arrow. Three males, I.2, III.1, and III.3, have the C337R variant. Two females, II.3 and III.4, carried the mutation. The parents of the index patient deceased before performing the study. The table shows demographic data and immunological studies. Abbreviations on the table: FP, Properdin; FB, Factor B; FD, Factor D; CH50, hemolytic activity of the Classical Pathway; AP50, hemolytic activity of the Alternative Pathway; NA, Not available, ND, no detectable. **(B)** FP serum levels were observed by Western blotting, confirming that three individuals (I.2, III.1, and III.3) were deficient and levels were decreased in a patient (III.4) that carried FP mutation. **(C)** Intracellular FP was detected by Western blotting using lysates from monocytes. Contrary to observed in plasma, FP was accumulated into monocytes from deficient (I.2, III.1 and III.3) and carrier (II.3) individuals, but not into monocytes from non-carrier individuals (II.4). The numbers below each line are the result of densitometry analysis and correction to the loading control, GAPDH. Each value was normalized with respect to the wild-type individual (II.4). FP: purified protein.

Immunological studies performed on the patient during remission revealed normal immunoglobulin levels, as well as complement components C3 and C4 within the normal range (Fig. 1A). The hemolytic activity of the classical pathway (CH50) was normal, but the AP activity (AP50) showed a slight reduction (Fig. 1A) suggesting an AP alteration (Fig. 1A). We then analyzed the levels of FP and complement factors B (FB) and D (FD) by in-house ELISAs. FB and FD showed normal levels but no FP could be detected (Fig. 1A). These findings confirmed the suspected diagnosis of FP deficiency. Sequencing of the coding region of the *CFP* gene resulted in the identification of the unreported sequence variant c.1009T > C (ENST00000247153.7) in exon 8 which caused an amino acid substitution of cysteine by arginine at position 337 (p.C337R) in the TSR5 domain. Full details of the methodology can be found in the Supplementary Data.

After the index patient's diagnosis, his maternal male cousin (III.3; Fig. 1A) showed a clinical picture compatible with meningococcal disease, confirmed by a positive hemoculture for *N. meningitidis* serogroup Y. All complement parameters were normal except for a low AP50 and undetectable FP, with *CFP* sequencing confirming the presence of the p.C337R variant (Fig. 1A).

Segregation studies revealed the variant had been inherited from the index patient's grandfather (I.2, Fig. 1A) (undetectable FP) while his aunt and female cousin were p.C337R carriers (II.3 and III.4, respectively; Fig. 1A). Of note, reduced FP levels were only observed in the III.4 (5.24 μg/mL; reference value: 17.5–45.5 μg/mL) (Fig. 1A). These results were also confirmed by Western blotting, as described in Supplementary Data (Fig. 1B).

FP deficiency is X-linked, which restricts symptomatology to hemizygotic males while heterozygotic females are usually unaffected. The finding of a compatible clinical and familial picture should raise suspicion of FP deficiency. However, biochemical and functional studies in specialized laboratories are mandatory to reach a diagnosis, and such difficulties most probably result in a global underdiagnosis of FP deficiency. This point is essential for carrier females, where circulating FP levels are variable even among females of the same family. In our case, there are two carrier females, II.3 with normal FP levels and III.4 with very low levels (Fig. 1A). This finding suggests that besides Mendelian segregation of the normal and abnormal FP alleles, random inactivation of the X chromosome greatly influences protein expression levels. The normal FP levels detected in the patient II.3 suggest major inactivation of the abnormal allele, while in case III.4, the low plasma FP levels are most likely the result of wild-type allele inactivation.

The index patient and his FP-deficient relatives, including his carrier cousin III.4, were immunized with a tetravalent polysaccharide meningococcal vaccine (Nimenrix, Pfizer, Spain) for protection against serotypes A, C, W-135, and Y, and with additional vaccine against serotype B (Bexero, GSK). They also received polyvalent pneumococcal and conjugated *Haemophilus influenzae* type b vaccines and when needed antibiotic prophylaxis.

To characterize this novel FP variant, monocytes from I.2, II.3, II.4, III.1 and III.3 were isolated and cultured, as described in Supplementary Data section. The cells were incubated under stimulated conditions with LPS, and FP was detected in the supernatant of cells from II.3 (35.9 ng/mL, carrier) and II.4 (37.7 ng/mL, wild-type) but not in the supernatant of cells from FP-deficient individuals (I.2, III.1, and III.3). Additionally, we analyzed intracellular FP in the patients’ monocytes and observed intracellular FP accumulation into the cells obtained from carrier and deficient individuals, as compared to cells from wild-type, healthy individuals (Fig. 1C). Interestingly, we detected several bands higher or lower than 53 kDa in a similar pattern in all individuals. We have not performed further investigations on the nature of each of the individual bands detected in the intracellular lysates but we assume that the two additional bands migrating at slightly higher and lower molecular weights (Fig. 1C) represent different maturation forms of the protein along the secretory pathway. However, we cannot discard that these bands are the result of unspecific detection by the polyclonal antibody. This is a limitation of the study that nevertheless does not significantly modify the conclusion that the c.1009T > C variant (p.C337R) in *CFP* is responsible for type I FP deficiency.

The T > C transition of exon 8 detected in our patients involves a TGT to CGT substitution resulting in a cysteine to arginine missense variant in the TSR5 domain. This change disrupts a disulfide bond formed between Cys337 and Cys376 which probably has severe conformational implications for FP folding and maturation. Monocyte cultures suggest a normal mRNA transcription of the c.1009T > C (p.C337R) allele but an aberrant protein folding of the protein that interferes with its secretion to the extracellular space. This novel variant is close to another pathogenic variant previously described in a Danish family with a type II FP deficiency.^3^ In that case, the authors found a single c.1028A > G transition in exon 8 resulting in a glutamine to arginine substitution at position 343 (p.Q343R). Monocytes from p.Q343R patients exhibit normal *CFP* mRNA and intracellular protein levels but extremely reduced FP plasma levels although *in vitro* LPS stimulation of p.Q343R monocytes triggers FP secretion.^3^ In striking contrast with Q343R, the C337R variant described by us completely fails to secrete FP in monocyte cultures even in the presence of LPS stimulation, which suggests that the integrity of the Cys337-Cys376 disulfide bridge is essential for FP folding and/or secretion.

This case report highlights the importance of considering FP deficiency as a potential cause of recurrent infections, particularly in the absence of abnormalities in routine immunological parameters. The specific individual risk of meningococcal infection in FP deficiency has been calculated at around 50%, a 250-fold increase over the baseline incidence of meningococcal infection in the general population.^4^ Genetic testing plays a crucial role in confirming the diagnosis, guiding management decisions, and offering appropriate counseling to affected individuals and their families. Early recognition and implementation of preventive measures, such as immunization and prophylactic antibiotics, can improve the clinical outcomes and quality of life for patients with FP deficiency, especially in individuals under 2 years of age in a family with FP deficient individuals.^5^

## Author contributions

F.C. and M.L-T designed the research. F.C., L.G-S., A.M.A., A.VdenR., and P.N. performed the experiments; M.I.B. and M.C.Y. provided the clinical data; F.C. wrote the original draft; L.G-S., A.L–L., A.VdenR, R.PdeD, P.N., and M.L-T. reviewed and edited the final version. All authors approved the final version of the manuscript.

## Conflict of interests

The authors declare no conflict of interests in this work.

## Funding

This study was funded by the Spanish Instituto de Salud Carlos III (ISCIII) and the 10.13039/501100008530European Regional Development Fund European Union (grant PI15-00255), by the Spanish Autonomous Region of Madrid (Complement II-CM network; S2017/BMD-3673), and F.C. was awarded a research fellowship by the Asociación Internacional de Familiares y Afectados de Lipodistrofias (AELIP).
