# Supplementary material for: Properdin deficiency associated with systemic meningococcal disease due to a novel p.Cys337Arg pathogenic variant
Source: Genes Dis. 2023 Oct 4;11(6):101134. doi: 10.1016/j.gendis.2023.101134 (PMC11295456; doi:10.1016/j.gendis.2023.101134)
Supplement: Multimedia component 1 [file mmc1.docx]

**MATERIALS AND MEHTODS**

**Patients and biological samples**

Blood samples were centrifuged at 2500 rpm, for 10 min, at a temperature of 4°C. Serum and EDTA-treated plasma were separated, aliquoted and stored at -80°C until use. DNA was extracted on a Maxwell® 16 Research Instrument System using a Maxwell1 LEV Blood DNA Purification Kit (Promega, Madison, WI, USA). The concentration and quality of the DNA were measured on a NanoDrop 1000 device (Saveen Werner, Limhamn, Sweden).

The study was conducted according to the guidelines of the Declaration of Helsinki, and approved by the Clinical Research Ethics Committee of La Paz University Hospital (project code PI-2276). All participants provided written informed consent for participation in the study and for the publication of their clinical and biochemical information.

**Genetic studies**

Genetic study of *CFP* was performed by polymerase chain reaction using DNA-specific primers, detailed in **supplementary Table 1**. Amplified products were purified from 2% low melting agarose gels using the QIAquick gel extraction kit (Qiagen Inc., Valencia, CA, USA). The sequencing reactions were performed in 10 μl, with 5–20 pmol of primer and using Big Dye terminator cycle sequencing v.3.1 (Applied Biosystems, Foster City, CA, USA). Sequencing products were precipitated in 65% ethanol, washed twice in 70% ethanol and analyzed on an automatic sequencing device (ABI-PRISM 3130, Applied Biosystems).

**Complement system studies**

Quantification of circulating C3, C4, IgG, IgA and IgM levels was performed by turbidimetry using the Attelica™ CH 930 System analyzer (Siemens-Healthineers, Spain). Circulating factor B (FB), factor D (FD) and FP were quantified using in-house ELISAs previously described by our group [1,2]. The functional activity of the CP and AP was measured by CH50 and AP50 hemolytic assay, respectively, according to standard procedures.

**Monocytes culture**

Peripheral blood mononuclear cells were isolated from 20 ml of EDTA-blood from FP-deficient individuals, female carriers, and normal individuals by Ficoll-Paque® density gradient centrifugation. Isolated cells were washed three times in RPMI medium 1640 (1X)+GlutaMAX^TM^-I (Gibco, Thermo Fisher Scientific, Waltham, MA, USA), and then resuspended in RPMI (1x10^6^ cells/ml) and transferred to 24-well plate (Costar®, Corning, Kennebunk, ME, USA) for 1h at 37ºC and 5% CO_2_. After incubation the non-adherent cells were removed by three washes with RPMI. The adherent cells were incubated with RPMI supplemented with 10% heat inactivated fetal bovine serum (FBS) (Thermo Fisher Scientific) for another 24h in the presence of lipopolysaccharide (LPS) (Invitrogen, Rockford, IL, USA) from *Escherichia coli* 026:B6 at a final concentration of 25 µg/ml. At the next day, the cells supernatants were collected and storage at -80ºC until FP quantification by ELISA. In order to analyze the intracellular FP by Western blot, adherent monocytes were lysed using RIPA lysis buffer and proteins were quantified with the BCA Protein Assay Kit (EMD Millipore Corp., Germany).

**Western blot analysis**

Extracellular and intracellular FP levels were analyzed by Western blot in plasma and monocytes lysates. Samples were separated on 10% polyacrylamide gels by SDS-PAGE and transferred to nitrocellulose membranes using iBlot 2 Dry Blotting System (Invitrogen). The membranes were incubated first with a specific goat polyclonal Ig GAHu/PPD (Nordic MUbio, Netherlands) at 1/1000 dilution, and followed by a rabbit anti-goat Igs/HRP conjugated (#P0449, Dako, Denmark) at 1/50000 dilution. As a loading control, GAPDH was detected using a GAPDH antibody (ZG003) (#39-8600, Invitrogen) and goat anti-mouse IgG-HRP conjugated (#107-6516, Bio-Rad, Hercules, CA, USA) at 1/1000 and 1/40000 dilution, respectively. Peroxidase activity was analyzed using ECL™ Select Western Blotting Detection Reagent (GE Healthcare Bio-Sciences, Piscataway, NJ, USA). UVITEC Alliance 1D MAX (UVITEC Cambridge) was used for image acquisition.

**REFERENCES**

1. Corvillo, F.; Bravo García-Morato, M.; Nozal, P.; Garrido, S.; Tortajada, A.; Rodríguez de Córdoba, S.; López-Trascasa, M. Serum Properdin Consumption as a Biomarker of C5 Convertase Dysregulation in C3 Glomerulopathy. *Clin. Exp. Immunol.* **2016**, *184*, 118–125, doi:10.1111/cei.12754.

2. Corvillo, F.; González-Sánchez, L.; López-Lera, A.; Arjona, E.; Ceccarini, G.; Santini, F.; Araújo-Vilar, D.; Brown, R.J.; Villarroya, J.; Villarroya, F.; et al. Complement Factor D (Adipsin) Levels Are Elevated in Acquired Partial Lipodystrophy (Barraquer-Simons Syndrome). *Int J Mol Sci* **2021**, *22*, 6608, doi:10.3390/ijms22126608.

**Supplementary Table 1. List of Primers Used in This Study**

| Exon | Forward Primer | Reverse Primer |
| --- | --- | --- |
| 1 | 5´-actcgatgctccctccac | 5´-tgtttgttactatatccccagcac |
| 2 | 5´-agttcctcctgcctctaggtt | 5´-ctcggtcagggatgtggt |
| 3 | 5´-cccaccacatccctgacc | 5´-aagaaagtgcccagttttgg |
| 4 | 5´-CTTACTGCGGACCTGGTGTT | 5´-AAGCAGACTGTCCCCAAATG |
| 5 | 5´-gaagcaaagagccctgaatg | 5´-ctggcagttccctgctgtaa |
| 6 | 5´-CTTACTGCGGACCTGGTGTT | 5´-AAGCAGACTGTCCCCAAATG |
| 7 | 5´-AAGGGTCTAGGGGCTGAGAG | 5´-ACTCGGCAAGGCAGATACC |
| 8 | 5´-ccctcattccttcctctgaa | 5´-ggtcttggtgggaaagtgag |
| 9 | 5´-AAGGGTCTAGGGGCTGAGAG | 5´-ACTCGGCAAGGCAGATACC |
| 10 | 5´-gtagccctcggcatatagca | 5´-gagatgctatcaccctacttttgg |
